# L-Glutamine alleviates osteoarthritis by regulating lncRNA-*NKILA* expression through the TGF-β1/SMAD2/3 signalling pathway

**DOI:** 10.1042/CS20220082

**Published:** 2022-07-06

**Authors:** Xiao Ma, Dechao Cai, Yakun Zhu, Yao Zhao, Xianbo Shang, Chen Wang, Haotian Zhang, Ashuai Bian, Haoran Yu, Wendan Cheng

**Affiliations:** 1 Department of Orthopedics, The Second Hospital of Anhui Medical University, Hefei, China; 2 Department of Orthopedics, Fuyang Hospital affiliated to Anhui Medical University, Fuyang, China

**Keywords:** chondrocytes, large intervening non-coding RNA, nuclear factor kappaB, osteoarthritis

## Abstract

Osteoarthritis (OA) is a heterogeneous condition characterized by cartilage degradation, subchondral sclerosis, and osteophyte formation, and accompanied by the generation of pro-inflammatory mediators and degradation of extracellular matrix. The current treatment for early OA is focused on the relief of symptoms, such as pain, but this treatment cannot delay the pathological process. L-Glutamine (L-Gln), which has anti-inflammatory and anti-apoptotic effects, is the most abundant amino acid in human blood. However, its role in OA has not been systematically studied. Therefore, the objective of this work was to explore the therapeutic effect and molecular mechanism of L-Gln on OA. *In vitro*, we found that L-Gln could up-regulate the expression of the long non-coding RNA *NKILA*, which is regulated by the transforming growth factor-β1/SMAD2/3 pathway, and inhibit the activity of nuclear factor-κB, thereby decreasing the expression of nitric oxide synthase, cyclooxygenase-2, and matrix metalloproteinase-13 (MMP-13). This led to a reduction in the generation of nitrous oxide, prostaglandin E-2, tumour necrosis factor-α, and degradation of the extracellular matrix (i.e. aggrecan and collagen II) in rat OA chondrocytes. Moreover, intragastric administration of L-Gln reduced the degradation of cartilage tissue and expression of MMP-13 in a rat OA model. L-Gln also relieved the clinical symptoms in some patients with early knee joint OA. These findings highlight that L-Gln is a potential therapeutic drug to delay the occurrence and development of OA.

## Introduction

Osteoarthritis (OA), a main cause of joint pain and disability in older individuals [1], affects the quality of life of more than 500 million people worldwide and increases health loads, creating visible implications for health-care systems and greater socioeconomic burdens [2,3].

OA mainly involves destruction of the cartilage tissue and whole joint and includes subchondral sclerosis, osteophyte formation, synovitis, and involvement of the infrapatellar fat pad [4,5]. Meanwhile, as a heterogeneous disease with a wide range of underlying pathways, OA is affected by age, body weight, inflammation, trauma, heredity, and other pathogenic factors; each pathogenic factor mainly affects different molecular pathways [3]. For instance, altered phenotypes of senescent cells can activate c-Jun N-terminal kinase (JNK) signalling by increasing cellular levels of reactive oxygen species (ROS), which worsens OA-associated phenotypes by activating chondrocyte apoptosis and extracellular matrix (ECM) degradation pathways [3,6]. Mechanical overloading can stimulate nuclear factor (NF)-κB signal transduction, accelerating the senescence of chondrocytes and occurrence of OA [3,7]. Thus, the pathogenesis of OA is extremely complicated and not completely elucidated yet [8]. Although the aetiology of OA is multifactorial, studies have revealed that the progression of OA is closely linked with inflammation [9], pro-inflammatory cytokines such as interleukin (IL)-1β, and tumour necrosis factor (TNF)-α play a pivotal role [10]. These cytokines can activate the NF-κB signalling pathway, in turn inducing chondrocytes and other articular tissue cells to produce matrix metalloproteinases (MMPs), cyclooxygenase-2 (COX-2), inducible nitric oxide synthase (iNOS), and other factors that lead to the inflammatory damage of chondrocytes and degradation of the extracellular matrix, resulting in destruction of articular tissues [4,11].

At present, the treatment of early OA includes non-drug based physical therapy such as exercise, weight loss, and avoid bearing heavy objects [12]. Treatment also includes anti-inflammatory and analgesic drug therapy, such as the oral or local use of non-steroidal anti-inflammatory drugs or intra-articular injection of prednisone and hyaluronic acid via the lateral of patellar ligament and inferior margin of patella [13,14]. Although these measures can provisionally relieve the symptoms of OA, they cannot prevent disease progression. With the deterioration of the disease, joint replacement surgery becomes crucial, but it can be very painful for the patient [15,16]. Thus, disease-modifying OA drugs (DMOADs) such as strontium ranelate, hydroxychloroquine, and TNF-α blockers, which can effectively relieve OA symptoms and improve the damaged cartilage structure, have become a new research direction [17,18]. However, these drugs are still in the preclinical research stage and are associated with major side effects and an unclear clinical efficacy in patients with OA [19]. Therefore, there is an urgent need to discover new agents for the treatment of OA.

L-Glutamine (L-Gln) is the most abundant and versatile conditionally essential amino acid in the body (i.e., it needs to increase the level of amino acid under some conditions, such as pressure) [20]. It plays a crucial role in cell composition, nitrogen exchange between organs, homeostasis, regulation of pH, and other physiological metabolic processes [20,21]. Such as, L-Gln can be used as a substrate for the synthesis of lipids, nicotinamide adenine dinucleotide phosphate (NADPH), proteins, and ribonucleotides, which are basic structural components of cells [21]. Free amines produced by nerve cells in the brain can be transported through the blood–brain barrier to the liver or kidneys in the form of Gln by means of the glutamate–glutamine cycle, where free ammonia is decomposed, released, and then discharged from the body [22]. L-Gln can also regulate the acid–base balance in the body by storing and releasing free ammonia thus maintaining pH homeostasis [23], and so on.

L-Gln has been extensively used for many years to treat different diseases. In a phase 3 clinical trial conducted by Niihara et al*.*, oral L-Gln was found to potentially increase the proportion of reduced nicotinamide adenine dinucleotide in sickle cell erythrocytes and reduce oxidative responses, thus reducing the pain crisis caused by sickle cell anaemia [24]. In dextran sodium sulphate (DSS)-induced colitis in mice, L-Gln can effectively treat gastrointestinal inflammatory diseases by inducing MAPK phosphatase (MKP)-1 to inactivate cytoplasmic phospholipase A2 (cPLA2) [25]. Supplementation with L-Gln in total parenteral nutrition after major abdominal surgery for pancreatitis can effectively prevent postoperative infections and complications, shorten hospital stay, and reduce the risk of death [26]. In addition, L-Gln can inhibit activation of the NF-κB pathway that is involved in inflammatory and apoptotic processes, and L-Gln exhaustion can lead to increased levels of pro-inflammatory mediators such as IL-1β, thus, attracting considerable clinical and research interest [27–29]. However, the effect L-Gln on OA and its specific molecular mechanism are still unclear.

Long non-coding RNAs (lncRNAs) have no protein coding function [30,31] but are involved in the formation of chromatin, regulation of signal transduction at the post-translational level, and regulation of gene expression [32,33]. Research has shown that LncRNA-*NKILA* (NF-κB interacting lncRNA) controls excessive activation of NF-κB by binding to the NF-κB/IκB complex to mask the phosphorylation site of IκB [34]. Transforming growth factor (TGF)-β1, a cytokine that regulates *NKILA*, binds to the TFG-β1 receptor, increasing the phosphorylation of SMAD2/3, which then accumulates in the nucleus, regulates the expression of *NKILA*, and prevents activation of NF-κB [35,36]. Therefore, as a negative feedback regulation mechanism, the expression of *NKILA* controls the “on/off” switch of NF-κB activation [34].

As established, L-Gln is involved in regulating the pathophysiological processes of various illnesses, but the efficacy and mechanism of L-Gln in OA remain unclear. The aim of the present study was to explore the potential therapeutic influences of L-Gln on the management of OA both *in vivo* and *in vitro*.

## Methods

### Primary chondrocyte culture

All animal experiments were approved by the experimental animal ethics committee of Anhui Medical University. Three-week-old Sprague-Dawley male rats (Animal Experimental Center of Anhui Medical University) were killed by spinal cord amputation and then the cartilage of the knee joint was peeled off. The samples were cut into 1 mm^3^ pieces and digested in 0.1% type II collagenase (Beyotime, China) for 4–6 h. The precipitate from this digestion was added to complete culture medium for rat chondrocytes (Cat# CM-R092, Procell, Wuhan, China, mainly containing DMEM/F12, transferrin, selenium, 15% foetal bovine serum, 1% penicillin, and streptomycin solution) and then cultured in a 37°C, 5% CO_2_ incubator. The cells were passaged with 0.25% trypsin (Beyotime) when they reached 80–90% confluence. To avoid phenotypic loss, only passage 1-3 chondrocytes were used.

### Cell viability analysis

According to a previous study, the administration of L-Gln (0, 5, 10, and 20 mM) to lipopolysaccharide (LPS)-stimulated human dental pulp cells has a concentration-dependent anti-inflammatory effect [28]. Therefore, chondrocytes (5 × 10^3^ cells/well) were seeded in 96-well plates and treated with the same concentrations of L-Gln (Biofroxx, Germany; 0, 5, 10, and 20 mM) for 24 and 48 h. Because IL-1β can reduce the viability of chondrocytes *in vitro* [37], the cells were pre-treated with 10 ng/ml IL-1β (PeproTech, Suzhou, China) for 2 h [38], followed by treatment with different concentrations of L-Gln (0, 5, 10, and 20 mM) for another 24 and 48 h. Cell viability was measured with the Cell Counting Kit-8 (CCK-8; Sigma-Aldrich, St. Louis, MO, U.S.A.), and absorbances were measured at 492 nm using a Rayto RT-6100 microplate reader.

### Enzyme-linked immunosorbent assay (ELISA)

According to the provided instructions, the concentrations of cytokines (TNF-α, IL-1β), prostaglandin E2 (PGE-2), and L-Gln in the supernatant of cultured chondrocytes and rat serum were determined using ELISA kits (R&D Systems), while the nitric oxide (NO) concentration was detected with Griess reagent. For ELISA and the Griess reagent, the absorbances were measured at 450 and 540 nm, respectively, using a Rayto RT-6100 microplate reader.

### Reverse transcription-quantitative polymerase chain reaction (RT-qPCR)

Total RNA was extracted from the chondrocytes using the RNA Easy Fast Tissue/Cell Kit (TIANGEN, China). The RNA was then reverse transcribed into cDNA, which was used as a template in qPCR using TB Green PreMix Ex Tap. The cycle threshold (Ct) values of all genes were obtained, normalized to that of *Gapdh* as the internal control, and the 2^−ΔΔCt^ algorithm was used to calculate the relative expression of the target genes. The primer sequences of all genes are shown in Table 1.

**Table 1:** Primer sequences used in RT-qPCR

Target genes	Forward primer (5′-3′)	Reverse primer(5′-3′)
Collagen II^1^	GAGTGGAAGAGCGGAGACTACTG	GTCTCCATGTTGCAGAAGACTTTCA
Aggrecan^1^	CTAGCTGCTTAGCAGGGATAACG	GATGACCCGCAGAGTCACAAAG
iNOS^2^	CAGGTGTTCCCCAGGTAGGTAG	CAGCATCCACGCCAAGAA
*Cox2*	GTTCCATTTGTGAAGATTCCTGTGT	CTCACTGGCTTATGCCGAAA
*Mmp13*	GGCCCTGAATGGGTATGACA	TGTCCCAAAGTGAACCCGCA
*Nkila* ^3^	CTGTCGGGGACTGGTGTATT	AATACACCAGTCCCCGACAG
*Gapdh* ^1^	GAAGGTCGGTGTGAACGGATTTG	CATGTAGACCATGTAGTTGAGGTCA

1 Bao J, Chen Z, Xu L, Wu L, Xiong Y. Rapamycin protects chondrocytes against IL-18-induced apoptosis and ameliorates rat osteoarthritis. Aging (Albany NY). 2020;12(6):5152–5167.

2 Zhao J, Nakahira K, Kimura A, Kyotani Y, Yoshizumi M. Up-regulation of iNOS protects cyclic mechanical stretch-induced cell death in rat aorta smooth muscle cells. Int. J. Mol. Sci. 2020;21(22).

3 Jia J, Zhang M, Li Q, Zhou Q, Jiang Y. Long noncoding ribonucleic acid NKILA induces the endoplasmic reticulum stress/autophagy pathway and inhibits the nuclear factor-k-gene binding pathway in rats after intracerebral hemorrhage. J. Cell Physiol. 2018;233(11):8839–8849.

### Western blotting

Chondrocytes (5 × 10^5^ cells/ml) were added to a Petri dish one day in advance. After the chondrocytes were pre-treated with 10 ng/ml IL-1β for 2 h followed by treatment with L-Gln (0, 5, 10, 20 mM) for another 24 h, the cells were suspended in RIPA buffer containing phosphatase and protease inhibitors (Beyotime), and supernatants were obtained. The protein concentrations were then determined using a BCA kit (Beyotime). Samples were heated at 100°C for 10 min after adding sample buffer (Beyotime). The proteins (10 μg total) were separated on a sodium dodecyl sulfate-polyacrylamide gel electrophoresis (SDS-PAGE) gradient gel with a concentration of 10–12% (voltage 70–120 V) and transferred to a polyvinylidene fluoride membrane (Millipore, U.S.A.). After blocking for 20 min with Western blotting rapid blocking solution (Beyotime), the membrane was incubated with primary antibodies at 4°C overnight, washed, and then incubated with a secondary antibody for 1 h. The Amersham ECL Plus western blotting reagent was used to detect bound antibody (ECL Plus Kit, GE Healthcare, U.K.), and images were observed using the AL600RGB gel imaging system and the Tanon5200 automatic luminous imaging system. The main antibodies used in this work were: COX-2 (ab179800, 1:1000, Abcam, Shanghai, China), aggrecan (DF7561, 1:1000, Affinity Biosciences, Lianyungang, China), collagen II (AF0135, 1:1000, Affinity), MMP-13 (AF5355, 1:1000, Affinity), GAPDH (ab8245, 1:3000, Abcam), p65 (AF7021, 1:1000, Affinity), p-p65 (AF2006, 1:1000, Affinity), IκBα (AF5002, 1:1000, Affinity), p-IκBα (AF2002, 1:1000, Affinity), SMAD2/3 (AF6367, 1:1000, Affinity), p-SMAD2 (AF3450, 1:1000, Affinity), and p-SMAD3 (AF3362, 1:1000, Affinity).

### Immunofluorescence (IF) staining

Chondrocytes (5 × 10^4^ cells/well) were inoculated in 24-well plates. After treatment with 10 ng/ml IL-1β for 2 h followed by treatment with L-Gln (0, 5, 10, or 20 mM) for another 24 h, the cells were fixed in 4% paraformaldehyde for 40 min. The cells were permeabilized with 0.4% Triton X-100 (Beyotime) for 25 min and blocked with 20% donkey serum (Shyuanye, China) for 45 min. The cells were incubated with antibodies (1:200) at 4°C for 8 h, followed by incubation for 30 min in the dark with the fluorescent secondary antibody (550037, Alexa Fluor 488, 1:500, ZBNBIO) at 25–30°C. Finally, the nuclei were stained with DAPI (Beyotime) for 6 min. Anti-fluorescence quenching agent was added dropwise. The images of p65, SMAD2/3, aggrecan, collagen II, and MMP-13 staining were captured with a laser confocal microscope (LSM8800, Carl Zeiss, Shanghai, China) and analysed by Image-Pro Plus 6.0 software.

### Animal experiments

Sprague-Dawley rats (male, 5-week-old, 240 ± 20 g,) were purchased from Beijing Weitong Lihua Experimental Animal Technology Ltd. (SCXK 2016-0006, Beijing, China). After 2 weeks of adaptive feeding, the rats were divided randomly into three groups (Control: *n*=6, OA: *n*=6, OA+L-Gln: *n*=6). In the light of the previous studies, the OA model was constructed by conducting anterior cruciate ligament transection and medial meniscus resection (ACLT+MMx) [38]. Briefly, each rat was anesthetized with 0.3% sodium pentobarbital (40 mg/kg, Sinopharm, Shanghai) combined with 1% lidocaine (Otsuka Pharmaceuticals, Shanghai), its right knee capsule was opened, the anterior cruciate ligament was transected, the medial meniscus was removed, and the articular capsule and skin were sutured. For the control group, the rats only had their knee capsules opened for joint exposure without ACLT+MMx. Four weeks after the surgery, intragastric administration of 0.9% NaCl at a dose of 0.25 g/kg was performed daily for the control and OA groups for 6 weeks. The OA+L-Gln group was administered L-Gln (iHerb, U.S.A.) intragastrically at a dose of 0.25 g/kg daily for 6 weeks. All animals were killed with the intraperitoneal injection of an overdose of sodium pentobarbital after intragastric administration of 6 weeks. Afterward, the knee joints and serum of the rats were collected for analysis. All rat trials were conducted in the Animal Experimental Center of Anhui Medical University and approved by the experimental animal ethics committee of Anhui Medical University.

### Histological staining

The knee joints were fixed with 5% paraformaldehyde and then decalcified with 10% ethylenediaminetetraacetic acid for 3 months. The sections were dehydrated and then embedded in paraffin. Sections (3–5 μm) were cut from the paraffin blocks and then stained with haematoxylin-eosin (H&E) and Safranin-O/Fast Green. The severity of OA was scored according to the Osteoarthritis Research Society International (OARSI) scoring system. The sections were imaged using a Tissue Quantitative system (TG, Austria).

### Immunohistochemical staining

The knee joint sections were dewaxed with xylene and dehydrated in ethanol, and then endogenous catalase was deactivated with H_2_O_2_. The sections were blocked with 10% donkey serum for 1 h; incubated with aggrecan, collagen II, and MMP-13 antibodies at 4°C for 6 h; incubated with the secondary antibody at 25–30°C for 30 min; and then visualized with a DAB kit (Beyotime). Haematoxylin was used for counterstaining. Images of the sections were captured by CaseViewer, and Image-Pro6.0 was used for quantitative assessment. The antibodies used in the study were aggrecan (DF7561, 1:100, Affinity), collagen II (AF0135, 1:100, Affinity), and MMP-13 (AF5355, 1:100, Affinity) antibodies.

### Screening of patients with early-stage OA and drug selection

The patients (*n*=47) with early knee OA diagnosed at the Second Affiliated Hospital of Anhui Medical University between October 2021 and January 2022 were included in the analysis. The inclusion criteria of the patients were (1) diagnosed with knee OA according to the guidelines for the diagnosis and treatment of OA in China [39]; (2) 18 years of age or older; (3) having Kellgren-Lawrence (K-L) grades 1-2 for the disease [40]; (4) no medications in the prior 2 weeks. Before and after the administration of 500 mg/day L-Gln (iHerb) for 4 weeks, the patients were respectively evaluated using the visual analogue scale (VAS), Western Ontario and McMaster Universities Arthritis Index (WOMAC), and Lequesne index as described previously [41,42]. Briefly, the degree of knee pain was evaluated by VAS (range: 0–10 cm). The OA severity and activity function of the knee joint was evaluated by the WOMAC and Lequesne index, respectively. WOMAC is composed of 24 items over 3 sections: 17 for physical function, 2 for stiffness, and 5 for pain. Participants rate the difficulty of each item every time using an 11-point numerical rating scale (NRS from 0 to 10) and the sum of all items results in a total score between 0 and 240, which represents the overall WOMAC score. The Lequesne index includes 11 items over 3 subscales: 5 for pain or discomfort, 2 for maximum walking distance with or without walking aids, and 4 for physical function disability. Each subscale has a score ranging from 0 to 8, and the sum of all items results in a total score between 0 and 24, which represents the overall Lequesne OA index score. In these three scales, the higher the score, the worse is the disease status of the patient. This work was authorised by the Medical Research Ethics Committee of the Second Affiliated Hospital of Anhui Medical University (No. YX2021-072), and all patients signed informed consent documentation.

### Statistical analysis

All measurable data are presented as the mean ± standard deviation (SD). The data were plotted using GraphPad Prism 6.02. One-way analysis of variance followed by Tukey’s post-hoc tests and Student’s *t*-tests were used to identify the statistical differences between the groups. All experiments were performed in triplicate and *P*<0.05 was deemed a statistically significant difference.

## Results

### Effect of L-Gln on chondrocyte viability

**Figure 1:**
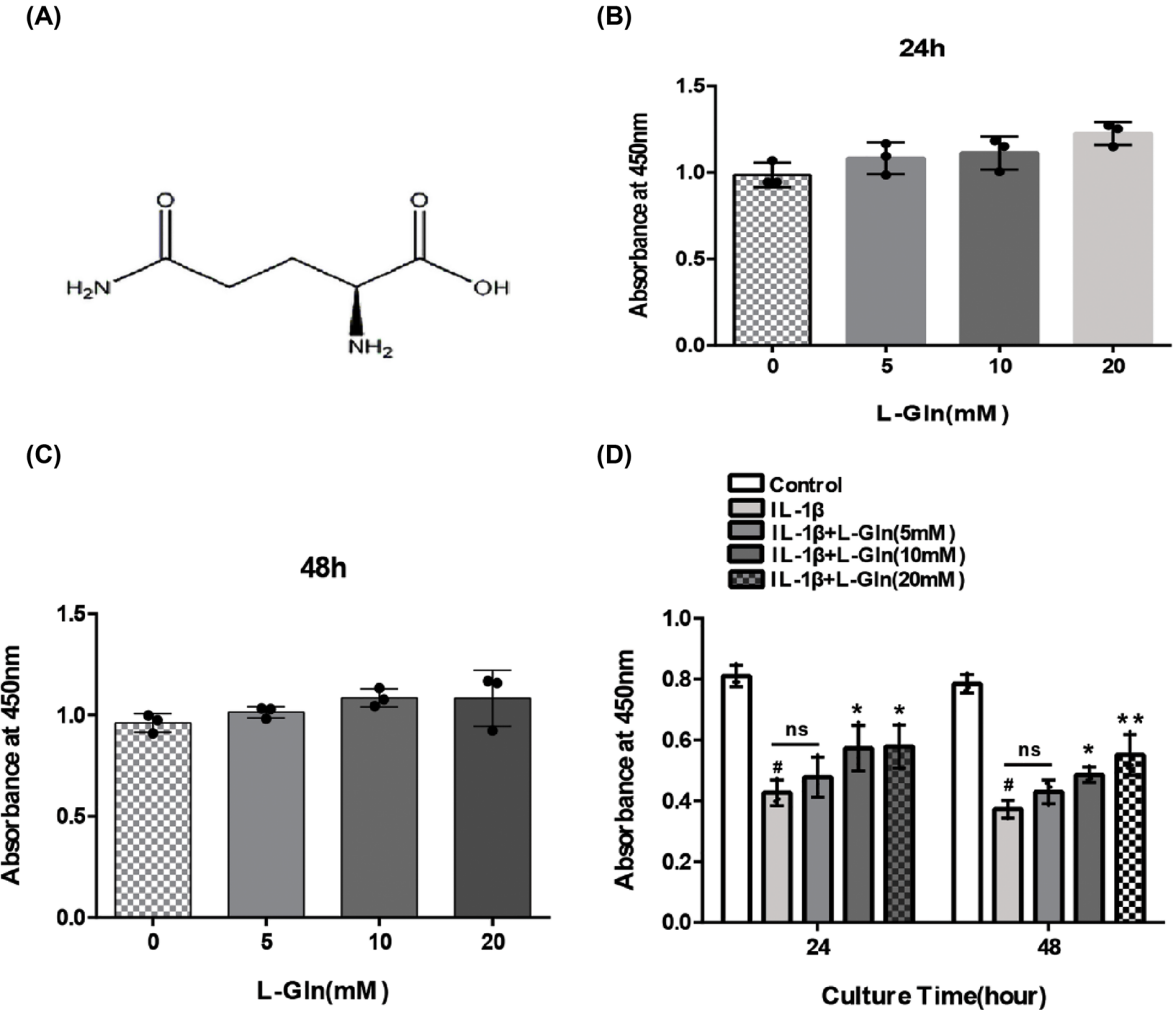
Effect of L-Glutamine (L-Gln) on chondrocytes viability (**A**) Molecular structure of L-Gln. (**B,C**) Chondrocytes were cultured with different concentrations of L-Gln (0, 5, 10, 20 mM) for 24 and 48 h. (**D**) Chondrocytes were pretreated with IL-1β, and cell viability was determined. #*P*<0. 05 versus the Control. *P<0.05, **P<0.01 versus the IL-1β group; *n*=3.

We first examined the effects of L-Gln (Figure 1A) on chondrocytes. The chondrocytes were treated with L-Gln (0, 5, 10, and 20 mM) for 24 and 48 h, and the CCK-8 assay was used to determine cell viability. The results revealed that L-Gln exhibited no toxicity in the chondrocytes up to 20 mM (Figure 1B,C). With respect to the decreased viability of chondrocytes treated with IL-1β, L-Gln could reverse this effect in a concentration-dependent manner (Figure 1D), indicating that L-Gln could enhance the viability of rat chondrocytes induced by IL-1β.

### L-Gln inhibits COX-2, iNOS, and MMP-13 expression, along with NO, PEG-2, and TNF-α release in rat OA chondrocytes

**Figure 2:**
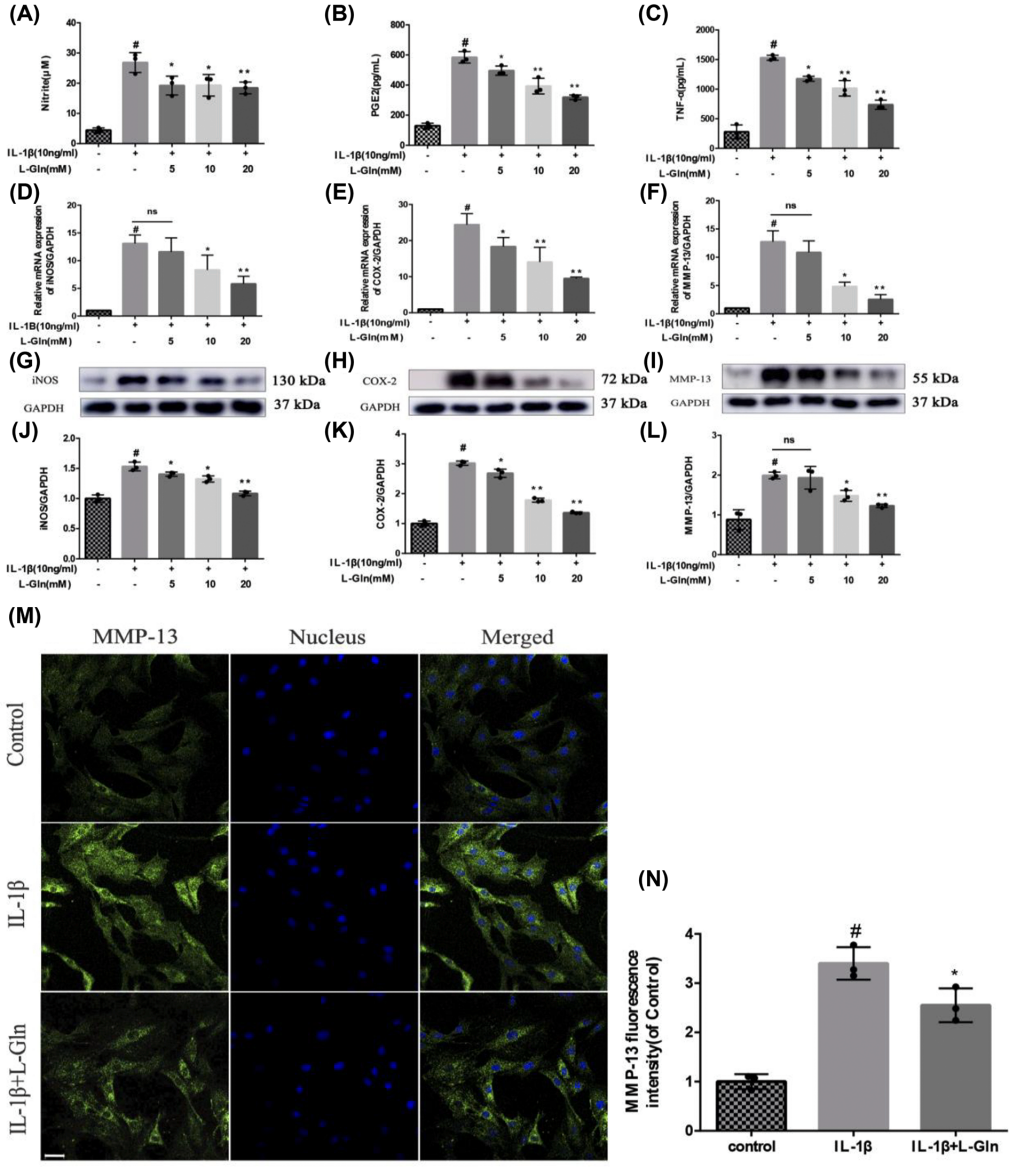
L-Gln inhibits IL-1β-induced cyclooxgenease-2 (COX-2), induced nitric oxide synthase (iNOS), and matrix metalloproteinase-13 (MMP-13) expression, and NO, prostaglandin E-2 (PEG-2), and tumour necrosis factor (TNF)-α release in rat osteoarthritis (OA) chondrocytes (**A**) Nitrite evaluated using the Griess test. (**B,C**) The levels of PGE-2 and TNF-α as determined by enzyme-linked immunosorbent assay. (**D–F**) The mRNA levels of iNOS, COX-2, and MMP-13. (**G–L**) The protein levels of iNOS, COX-2, and MMP-13. (**M,N**) MMP-13 as determined by immunofluorescence and analyzed with Image-Pro6.0; scale bar = 50μm. #*P*<0.05 versus the control group. *P<0.05, **P<0.01 versus the IL-1β group; *n*=3.

To explore the effect of L-Gln on OA chondrocytes, the cells were pre-treated with 10 ng/ml IL-1β for 2 h followed by treatment with L-Gln (0, 5, 10, or 20 mM) for another 24 h. The results showed that IL-1β markedly increased the expression levels of iNOS, MMP-13, and COX-2 in addition to increasing the production of NO, TNF-α, and PGE-2 in the chondrocytes. However, L-Gln reversed these effects with the most significant outcome observed at 20 mM L-Gln (*P*<0.01) (Figure 2A–L). Therefore, 20 mM L-Gln was used for the IF staining. The IF results showed that this concentration of L-Gln could inhibit the expression of MMP-13 in rat OA chondrocytes (Figure 2M,N). These results revealed that L-Gln could effectively reduce the production of pro-inflammatory factors in OA chondrocytes

### L-Gln inhibits the degradation of extracellular matrix (collagen II, aggrecan) in rat OA chondrocytes

**Figure 3:**
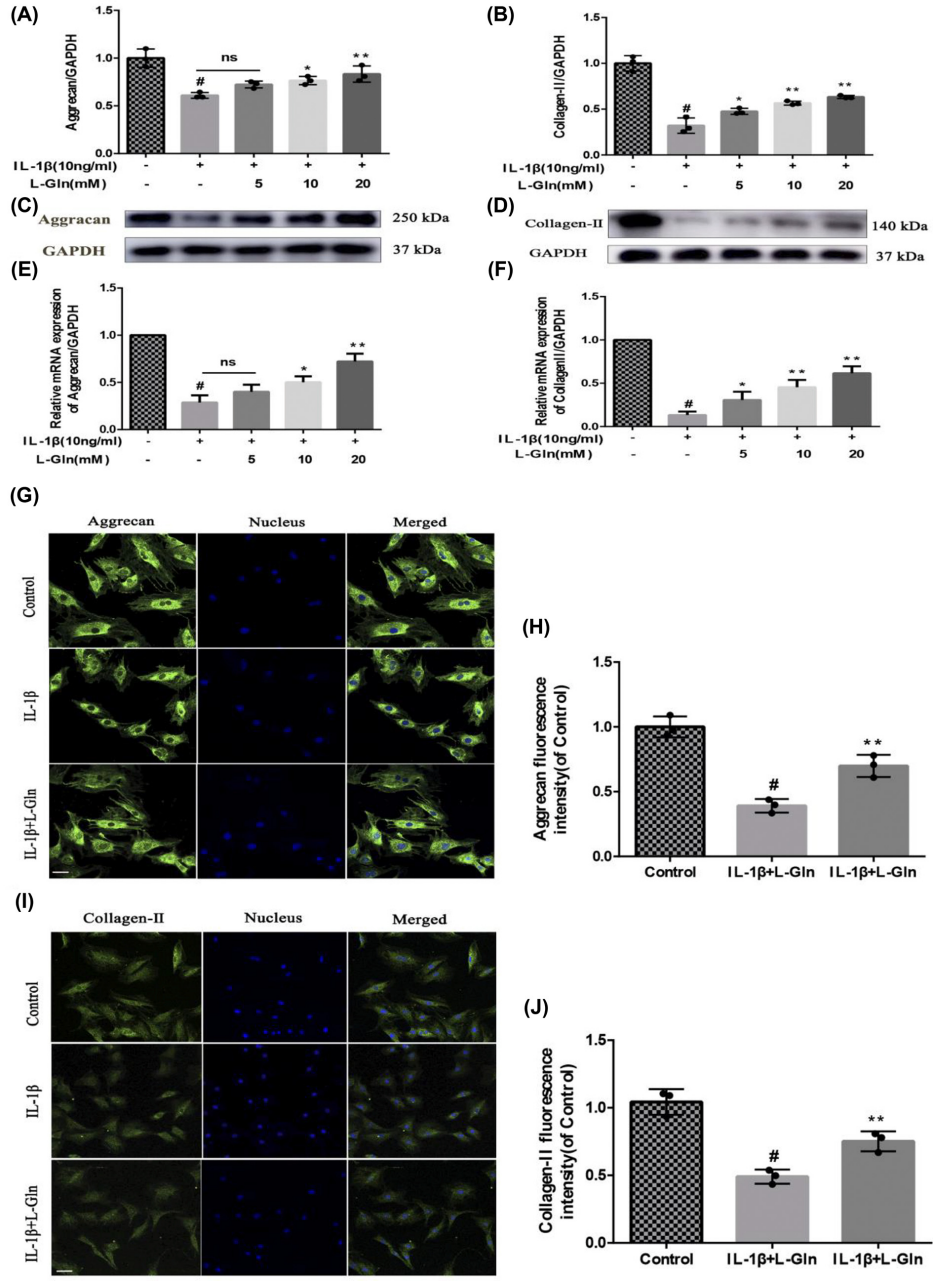
L-Gln inhibits the degradation of extracellular matrix in rat OA chondrocytes (**A–D**) Protein levels of Collagen II and Aggrecan. (**E,F**) mRNA levels of Aggrecan and Collagen II. (**G–J**) Fluorescence intensity of Aggrecan and Collagen II as determined by immunofluorescence and analyzed with Image-Pro 6.0; scale bar = 50 μm. #*P*<0.05 versus the Control. *P<0.05, **P<0.01 versus the IL-1β group; *n*=3.

To further investigate the effect of L-Gln on extracellular matrix proteins (collagen II and aggrecan) in OA chondrocytes, we pre-treated the cells, as previously described, with 10 ng/ml IL-1β for 2 h followed by treatment with L-Gln (0, 5, 10, or 20 mM) for 24 h. We found that L-Gln alleviated the degradation of collagen II and aggrecan caused by IL-1β both at the mRNA and protein levels (*P*<0.01) (Figure 3A–F). Meanwhile, IF also showed that L-Gln (20 mM) notably inhibited the degradation of OA chondrocyte extracellular matrix (Figure 3G–J), which was consistent with the results of RT-qPCR and Western blotting. In brief, these results indicated that L-Gln could effectively attenuate the catagenesis of extracellular matrix in OA chondrocytes.

### L-Gln inhibits over-activation of NF-κB in rat OA chondrocytes

**Figure 4:**
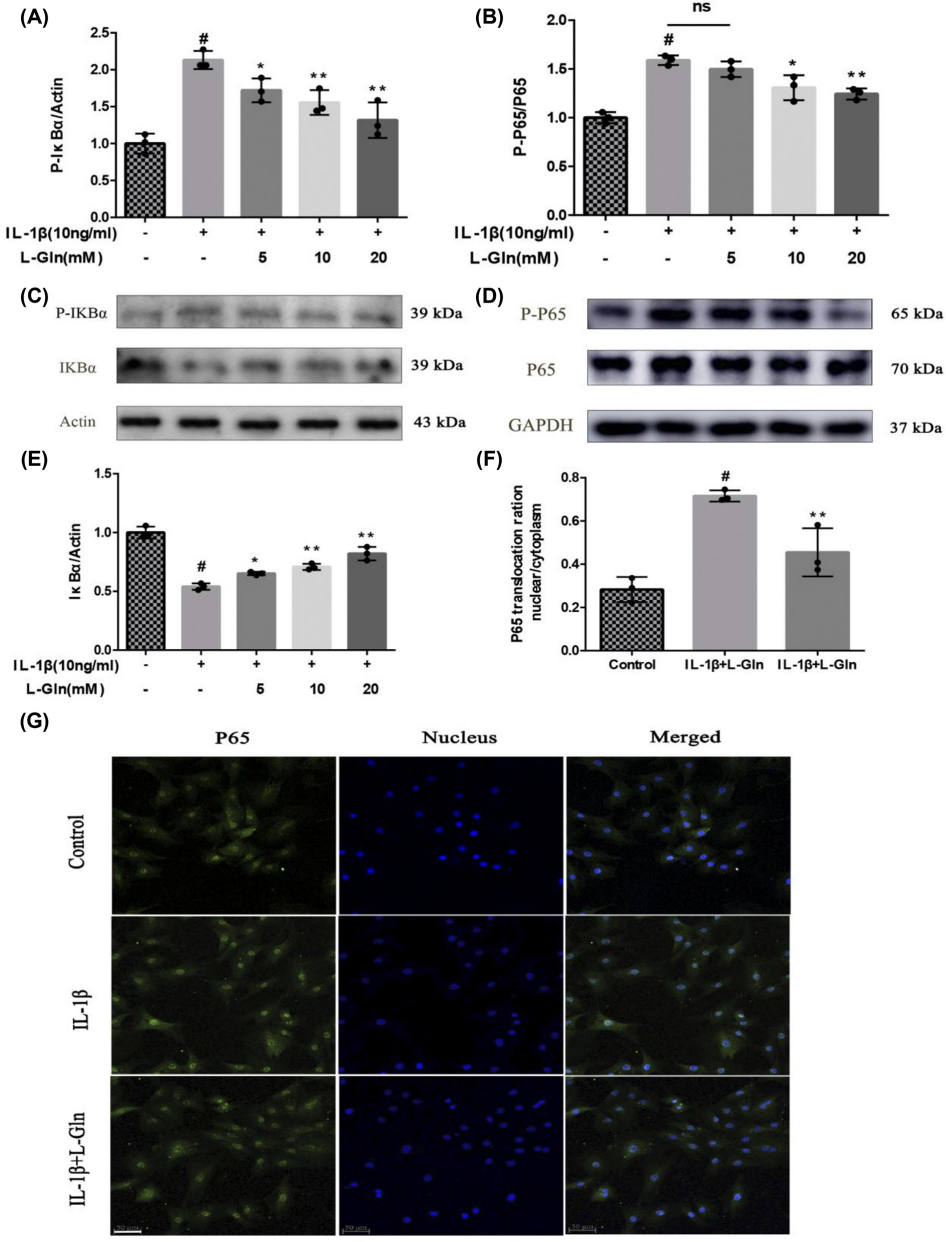
L-Gln inhibits over-activation of nuclear factor-κB (NF-κB) in rat OA chondrocytes (**A–E**) Protein levels of p65, p-IκB, IκB, and p-P65. (**F,G**) Nuclear translocation of p65 visualized by immunofluorescence and quantified with Image-Pro6.0; scale bar = 50 μm. #*P*<0.05 versus the control group. *P<0.05, **P<0.01 versus the IL-1β group; *n*=3.

NF-κB, which is associated with inflammation, plays an indispensable role in the progression of OA, and nuclear translocation of p65 is the core feature of NF-κB activation [43]. As displayed in Figure 4A–E, the phosphorylation of p65 and IκBα, and the degradation of IκBα markedly increased after chondrocytes were exposed to IL-1β (*P*<0.01), but after L-Gln treatment, p-p65, p-IκBα, and the degradation of IκBα were significantly decreased (*P*<0.01). Meanwhile, p65 IF demonstrated that IL-1β could induce p65 accumulation in the nucleus, but 20 mM L-Gln could inhibit this change (Figure 4F,G). These outcomes show that L-Gln can weaken the over-activation of NF-κB and potentially alleviate OA inflammation.

### L-Gln inhibits NF-κB over-activation in rat OA chondrocytes by regulating *NKILA* induced by the TGF-β1/SMAD2/3 pathway

**Figure 5:**
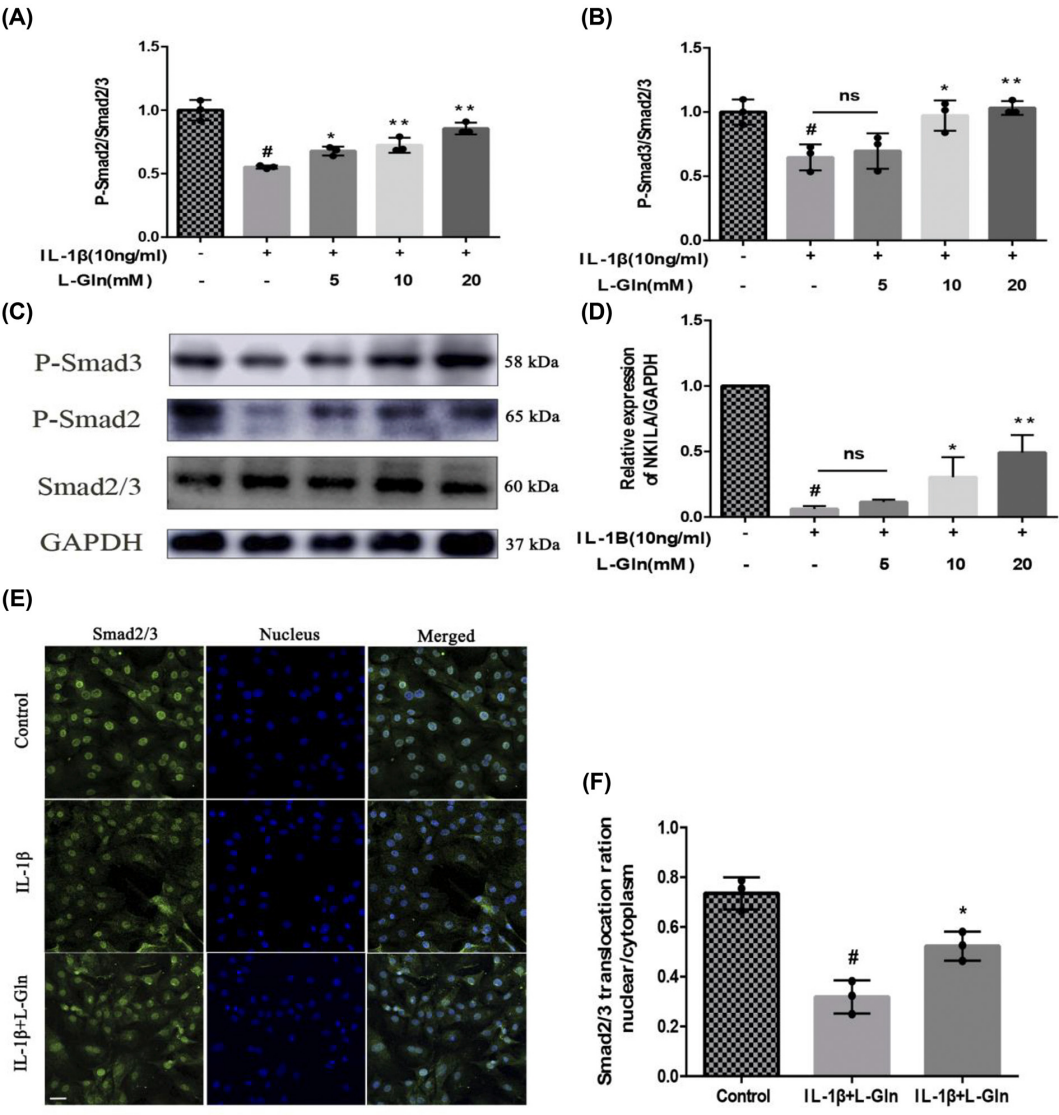
L-Gln inhibits NF-κB over-activation in rat OA chondrocytes by regulating TGF-β1/SMAD2/3 pathway-induced NKILA (**A–C**) Protein levels of p-SMAD2, SMAD2/3, and p-SMAD3. (**D**) RNA level of NKILA. (**E,F**) Nuclear translocation of SMAD2/3 visualized by immunofluorescence and quantified with Image-Pro6.0; scale bar = 50 μm. #*P*<0.05 versus the control group. *P<0.05, **P<0.01 versus the IL-1β group; *n* =3.

LncRNA-*NKILA* inhibits NF-κB over-activation by binding to the IκB/NF-κB complex, and TGF-β1-induced nuclear translocation of Smad2/3 is one of the key factors regulating *NKILA* expression [43,44]. Therefore, we evaluated the inhibitory effect of L-Gln on NF-κB by detecting the phosphorylation of Smad2/3 and the expression of *NKILA* in rat OA chondrocytes. As shown in Figure 5A–D, the phosphorylation of Smad2/3 and expression of *NKILA* were decreased observably after the chondrocytes were exposed to IL-1β; however, the levels of both p-Smad2/3 and *NKILA* gradually approached those of the Control cells after treatment with L-Gln (*P*<0.01). Meanwhile, IF of Smad2/3 also indicated that intranuclear localization of Smad2/3 in chondrocytes was attenuated by IL-1β, and L-Gln (20 mM) could reverse this change (Figure 5E,F). Thus, the results showed that L-Gln can up-regulate the expression of *NKILA* through the TGF-β1/Smad2/3 pathway to attenuate NF-κB over-activation in OA chondrocytes.

### L-Gln alleviates rat OA *in vivo*

**Figure 6:**
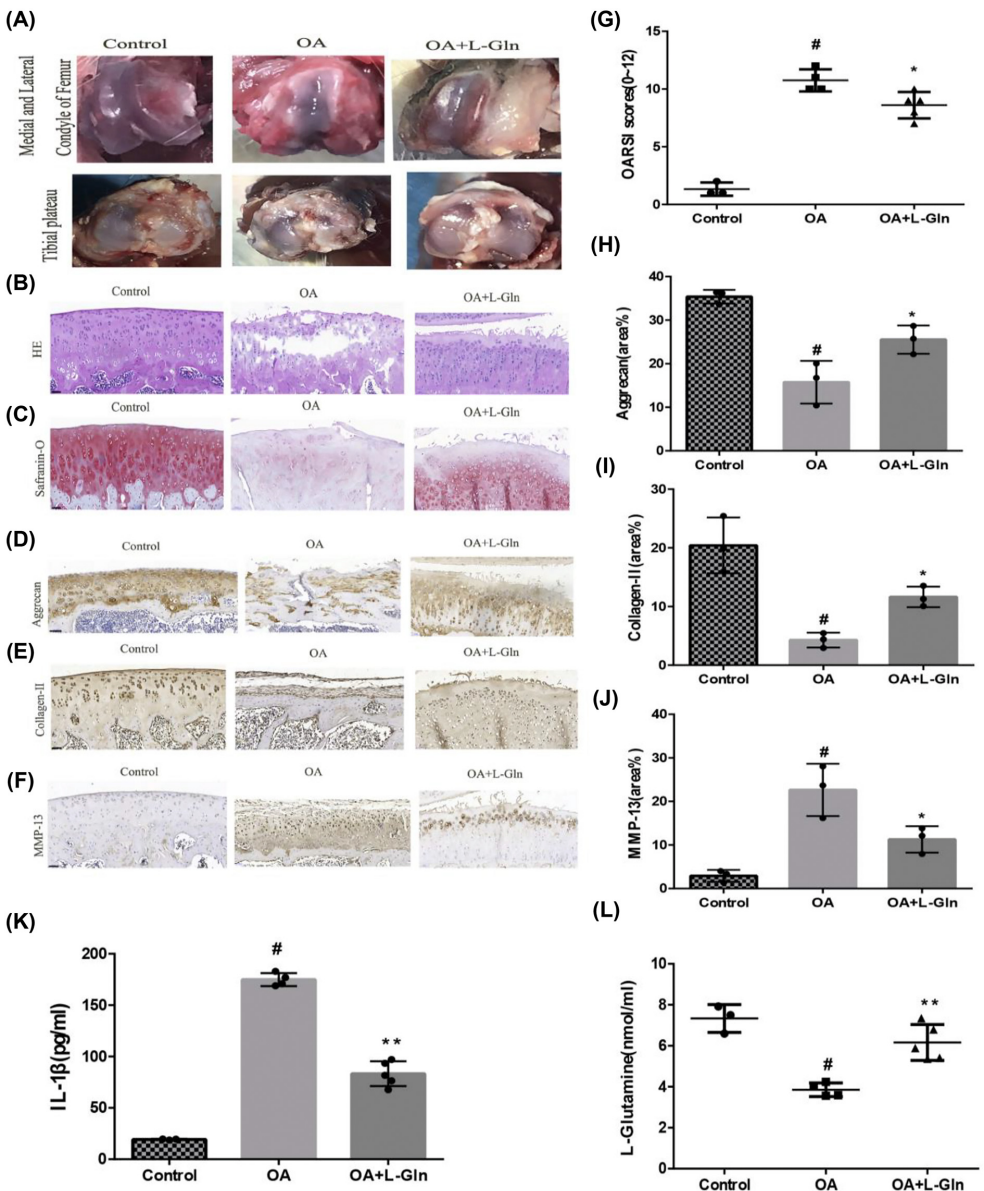
Intragastric administration of L-Gln can delay the development of OA in rats (**A**) The medial and lateral femoral condyle and tibial plateau of rats. (**B**) H&E staining. (**C**) Safranin-O/Fast Green staining. (**D–F**) Immunohistochemical staining of MMP-13, Aggrecan, and Collagen II. (**H–J**) Quantification with Image-Pro6.0; scale bar = 50 μm. (**G**) OARSI score. (**K, L**) The serum levels of IL-1β and L-Gln. #*P*<0.05 versus the control group. *P<0.05, **P<0.01 versus the OA group; Control: *n*=3, OA: *n*=4, OA+L-Gln: *n*=5.

To further explore the effect of L-Gln on OA, an OA model was established in rats by ACLT+MMx, as described above. Four weeks after ACLT+MMx, the rats were intragastrically administered 0.25 g/kg L-Gln every day for 6 weeks, and all rats were euthanised. The knee joints were observed, graded by OARSI, examined by histopathological (H&E, Safranin-O/Fast-green) and immunohistochemical staining. Anatomical observations of the knee joint showed that, compared with that of the Control group, there were severe defects and even exfoliation of the cartilage in the medial and lateral condyle of the femur and tibial plateau, thinning of articular cartilage, and exposure of subchondral bone in the OA group; the degree of destruction of the knee cartilage was observably reduced in the OA+L-Gln group (Figure 6A) and the OARSI score of the OA+L-Gln group was also observably lower than that of the OA group (*P*<0.05) (Figure 6G). H&E staining also revealed that the cartilage surface of the OA knee joints was discontinuous, the cartilage was thinner, the tidal line was disordered, and the matrix was significantly reduced, but the OA+L-Gln knee joints had markedly improved structural integrity of the articular cartilage (Figure 6B), which was consistent with the results of Safranin-O/Fast-green staining (Figure 6C). Immunohistochemical staining revealed that the deposition of aggrecan and collagen II was markedly reduced, and MMP-13 was increased in the OA group (*P*<0.05). Intriguingly, treatment with L-Gln prominently attenuated these changes (*P*<0.05) (Figure 6D–F,H–J). Chien et al*.* showed that IL-1β plays a crucial role in OA progression [45]. This is consistent with our results from the rat serum ELISA, which showed that the production of IL-1β in the OA group was increased relative to that in the Control group, and L-Gln could remarkably decrease this change (Figure 6K). Geir et al*.* revealed that glutamine depletion can result in the levels of pro-inflammatory mediators improved such as IL-1β [46]. Our results also showed that the level of L-Gln in the OA group was significantly lower than that in the Control group (*P*<0.05), while the level in the OA+L-Gln group was higher (Figure 6L). The results of ELISA indicated that the intragastric administration of 0.25 g/kg/day of L-Gln for 6 weeks could notably increase serum L-Gln levels, thus, inhibiting the production of IL-1β in rat serum. In short, these observations revealed that L-Gln could significantly alleviate cartilage degeneration, enhance the structural integrity of articular cartilage, and delay OA progression in the rat model.

### L-Gln partially improves symptoms in patients with early OA

**Figure 7:**
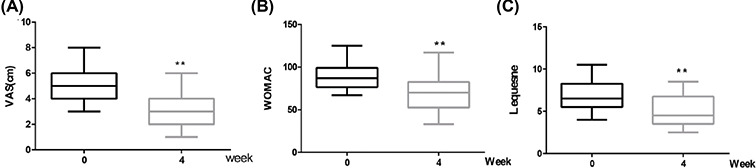
L-Gln improves the symptoms of some patients with early OA (**A**) visual analogue scale (VAS), (**B**) WOMAC and (**C**) Lequesne scores at 0 and 4 week after oral administration of L-Gln, respectively. **P<0.01 versus week 0; *n*=41.

To further probe the effects of L-Gln on OA, we conducted a 4-week clinical trial. According to K-L grade [40], 47 patients with early knee OA (K-L-I/II) were selected from October 2021 to January 2022. This included 23 cases (6 men, 17 women) of K-L-I, and 26 cases (6 males, 20 females) of K-L-II. The average age was 57.5 ± 10.4 years. Forty-one cases experienced return visits, a rate of 87.2%. Patients were administered 500 mg/day oral L-Gln. The degree of knee pain was evaluated by VAS; the severity of OA before administration of L-Gln and the therapeutic effects after 4 weeks of L-Gln administration were assessed using the WOMAC and Lequesne index (Figure 7A–C). After taking 500 mg/day L-Gln for 4 weeks, the WOMAC score in 34 cases and the Lequesne score in 36 cases decreased to varying degrees (*P*<0.01), the distribution of scores was close to a Gaussian distribution, with OA improvement rates of 82.9% and 87.8%, respectively (Table 2). It is worth mentioning that in 5 out of 41 cases, OA symptoms were observably improved and the WOMAC and Lequesne scores were markedly decreased after taking L-Gln for 4 weeks, despite the ineffectiveness of previous Glucosamine administration. These effects illustrate that L-Gln has a positive influence on patients with early knee OA, reducing knee symptoms, improving joint mobility, and enhancing quality of life in some patients.

**Table 2:** WOMAC, Lequesne Scores, and OA Improvement rate

Groups	WOMAC	Lequesne
Before	89.24 ± 14.14	7.11 ± 2.13
4 weeks	68.59 ± 18.52	4.90 ± 1.98
OA improvement rate (%)	82.9	87.8

## Discussion

OA, a common form of whole joint damage, involves the meniscus, synovial membrane, and infrapatellar fat pad [5]. The main symptom of OA is joint pain, which can lead to disability [13,47]. Currently, there is an increasing demonstration that OA is a dynamic process prompted by the imbalance of joint damage and remodelling, in which the excessive production of pro-inflammatory mediators resulting in chondrocyte apoptosis and matrix degradation is a major cause of OA [48,49]. The treatments recommended by international guidelines for the treatment of OA are only meant to improve symptoms and cannot delay the pathological process; the long-term efficacy and safety of these treatments are also uncertain [12,50]. Recently, anti-inflammatory amino acid drugs have attracted attention for the treatment of OA owing to the occurrence of fewer side effects [51].

Herein, we illustrate for the first time that L-Gln can effectively delay articular cartilage degeneration and reduce inflammation in OA. These effects occur partly through the up-regulation of lncRNA-*NKILA* expression by the TGF-β1/Smad2/3 pathway and the inhibition of over-phosphorylation of NF-κB. Intragastric administration of L-Gln also significantly reduced the degradation of cartilage tissue and peripheral inflammation in rats. In addition, oral L-Gln administered to some patients with early OA significantly improved symptoms. This indicates that L-Gln may be a treatment for OA. Our results also provided evidence for the potential, broad clinical application of DMOADs.

The inflammatory mediators IL-1β and/or TNF-α are the main factors promoting the destruction of cartilage and degeneration of articular cartilage in OA [52,53]. However, IL-1β and TNF-α are not always detectable in the cartilage of patients with OA [54]. In fact, all OA joint tissues release inflammatory mediators, and IL-1β and/or TNF-α are generally used as pro-inflammatory stimuli *in vitro* to study inflammatory responses in OA [5]. For instance, under IL-1β stimulation, chondrocytes can produce and release NO, PGE-2, and TNF-α, whereas the meniscus liberates MMP-3/13 and the infrapatellar fat pad releases high levels of IL-6/8 [5,55]. This in turn results in the apoptosis of chondrocytes and degradation of the extracellular matrix, which leads to a vicious cycle of joint tissue degradation and inflammation that further contributes to the progression of OA [56,57].

In the present study, NO, PGE-2, and TNF-α were markedly up-regulated in rat chondrocytes stimulated with IL-1β (*P*<0.01), but after L-Gln treatment, the levels of NO, PGE-2, and TNF-α were significantly reduced (*P*<0.01) (Figure 2A–C). Moreover, the viability of chondrocytes stimulated with IL-1β was increased (Figure 1D). Therefore, our *in vitro* results prove that L-Gln could preserve chondrocytes by inhibiting these inflammatory factors.

NF-κB, part of a classical inflammatory signalling pathway, is a transcription factor complex widely expressed on the surface of nuclear membranes, and it plays a crucial role in the occurrence and development of OA [58,59]. Under the effect of pro-inflammatory mediators such as IL-1β, the activity of NF-κB in chondrocytes is up-regulated and the phosphorylation of p65 and IκBα and the degradation of IκBα are increased, thereby inducing the expression of inflammatory mediators such as COX-2, iNOS, and MMPs [60]. As revealed in prior studies, iNOS inhibits the synthesis of aggrecan and collagen II by inducing the excessive accumulation of NO, and COX-2 accelerates the decomposition of aggrecan and collagen II by inducing the production of PGE-2 [55]. MMPs participate in chondrocyte degradation and extracellular matrix destruction [61]. In particular, the expression of MMP-13 is dramatically increased during the development of OA, and it is known that MMP-13 is a member of the key subfamily of metalloproteinases involved in cartilage matrix decomposition [55,62]. At present, there is increasing evidence that inhibiting COX-2, iNOS, and MMP-13 could delay the occurrence and development of OA [63,64], and that NF-κB p65-specific siRNA could reduce the production of inflammatory factors induced by IL-1β [65]. Therefore, targeted blockade of NF-κB is conducive to delaying the pathological process of OA.

Herein, we examined the anti-inflammatory effect of lncRNA-*NKILA*, which inhibits the NF-κB signalling pathway, on OA. Our results showed that L-Gln could partially inhibit p65 and IκBα phosphorylation, IκBα degradation, and the translocation of p65 from the cytoplasm to the nucleus (Figure 4). This maintained chondrocyte phenotypes by decreasing the expression of COX-2, iNOS, and MMP-13 (Figure 2D–L) and delayed IL-1β-induced cartilage matrix degradation in OA chondrocytes (Figure 3). This was consistent with the results of the intragastric administration of L-Gln *in vivo* (Figure 6). In addition, when oral L-Gln was administered to some patients with early OA, the symptoms of OA were significantly improved (Figure 7). Thus, L-Gln can reduce the expression of COX-2, iNOS, and MMP-13 by inhibiting NF-κB overactivity in OA, thus, delaying the progression of OA.

*NKILA* was recently found to be up-regulated by pro-inflammatory cytokines in breast cancer [66], and there is increasing evidence that NKILA can act as an inhibitor of NF-κB in cellular inflammation and regulate the body’s inflammatory response [67]. However, it is not clear whether L-Gln inhibits the over-activation of NF-κB by regulating *NKILA* in OA chondrocytes. In this work, we verified that the expression of *NKILA* induced by p-Smad2/3 was significantly inhibited in rat OA chondrocytes (*P*<0.01), which was consistent with the findings of Yu et al. [68]. However, after L-Gln treatment, the levels of p-Smad2/3 and *NKILA* gradually increased in a concentration-dependent manner (Figure 5A–E). Therefore, L-Gln can regulate the expression of *NKILA* by up-regulating the phosphorylation of Smad2/3 in OA chondrocytes.

Previous research has also determined that *NKILA* has a high affinity for IκB [69]. By binding to IκB, *NKILA* and NF-κB/IκB form a new complex that can inhibit the phosphorylation of IκB, thereby reducing the activation of NF-κB [34,43]. It was confirmed in this work that *NKILA* is a regulatory factor upstream of NF-κB, which is affected by the level of p-Smad2/3, and that L-Gln can regulate the expression of *NKILA* by increasing Smad2/3 phosphorylation, thereby inhibiting the levels of p-p65 and p-IκBα in OA chondrocytes (Figure 5).

The present study had a few limitations. Owing to financial and time constraints, the lack of an *NKILA* gene silencing model was one limitation. The small sample size of clinical patients was another limitation. Moreover, the concrete interaction between L-Gln and TGF-β1 and the molecular mechanism involved were not elucidated in detail.

In summary, this study revealed the anti-inflammatory and protective effects of L-Gln on OA, providing potential evidence for L-Gln as a DMOAD for future clinical applications.

## Clinical perspectives

- Osteoarthritis severely reduces quality of life, and there is currently no effective treatment beyond symptom management. Disease-modifying osteoarthritis drugs can alleviate osteoarthritis, but these drugs have major side effects. Recent studies point to the potential of amino acids such as L-glutamine to alleviate inflammation in osteoarthritis, but the therapeutic effect and mechanism remain unclear.
- The present study shows that L-glutamine can regulate the expression of the long non-coding RNA *NKILA* by increasing p-SMAD2/3 to prevent the over-activation of NF-κB, thus protecting chondrocytes *in vitro* and alleviating the production of inflammatory cytokines and osteoarthritis symptoms in a rat model *in vivo*. Moreover, in a small clinical trial, some patients with early knee osteoarthritis experienced marked symptom reduction after 4 weeks of oral L-glutamine.
- These findings highlight L-glutamine as a new safe and effective clinical treatment to delay the pathogenesis of osteoarthritis.

## Data Availability

The authors declare that all the data supporting the findings of this study are available within the article and its Supplementary Information files.

## Abbreviations

COX-2: cyclooxgenease-2
ELISA: enzyme-linked immunosorbent assay
H&E: haematoxylin-eosin
iNOS: induced nitric oxide synthase
MMP-13: matrix metalloproteinase-13
OA: osteoarthritis
PEG-2: prostaglandin E-2
TNF: tumour necrosis factor
